# The Y-Pelvic Osteotomy in Treating Bladder Exstrophy: A Surgical Technique

**DOI:** 10.7759/cureus.30520

**Published:** 2022-10-20

**Authors:** Sattar Alshryda, Ibrar Majid, Ghadir Jaber, Diary Mohammad, Mamoun Al Marzouqi

**Affiliations:** 1 Pediatric Orthopedics and Trauma, Al Jalila Children's Speciality Hospital, Dubai, ARE; 2 Orthopedic Surgery, Mohammed Bin Rashid University of Medicine and Health Sciences, Dubai, ARE; 3 Pediatric Orthopedics, Al Jalila Children's Speciality Hospital, Dubai, ARE; 4 Pediatric Surgery, Al Jalila Children's Speciality Hospital, Dubai, ARE; 5 Pediatric Surgery and Urology, Al Jalila Children's Speciality Hospital, Dubai, ARE

**Keywords:** cloacal exstrophy, epispadias and bladder exstrophy, pelvis osteotomy, persistent cloaca, bladder reconstruction

## Abstract

Bladder exstrophy (BE) is a rare congenital anomaly caused by an embryological defect in the closure of the abdominal wall. It comprises a spectrum of defects about severity, including epispadias in the mildest form and cloacal exstrophy in the worst.

Surgical correction is required to achieve urinary continence, maintain normal renal function, achieve secured abdominal wall closure, and create cosmetically and functionally satisfactory genitalia. Iliac bone osteotomy is considered essential to achieve the above goals in most patients by reducing the tension of the closed abdominal wall layers, particularly when present late in infancy.

Several types of pelvic iliac bone osteotomy have been described to aid bladder and cloacal exstrophy closure. They can be grouped into posterior iliac osteotomy, anterior iliac osteotomy, oblique (also called diagonal) iliac osteotomy, and a combination of posterior and anterior iliac osteotomy.

We described here the Y-pelvic osteotomy, which was developed by the Manchester Orthopaedic Group in the United Kingdom. It has the advantage of anterior and posterior osteotomies but also has less risk to the neurovascular structures, less blood loss, and ease of surgical technique. The osteotomy was named the Y-pelvic osteotomy due to the morphological shape it resembles.

## Introduction

Bladder exstrophy (BE) is a rare congenital anomaly caused by an embryological defect in the closure of the abdominal wall. There is a wide spectrum of severity. Epispadias represents the mildest form of exstrophy whereas cloacal exstrophy is the most severe. 

The condition is uncommon. The reported incidence of BE ranges from 1 in 30,000 to 1 in 50,000. Cloacal exstrophy is even rarer with a reported incidence of 1 in 200,000 to 1 in 400,000. Males are affected two to three times more often than females [1-4].

Surgical correction is required to achieve urinary continence, maintain normal renal function, and create cosmetically and functionally satisfactory genitalia [5]. Iliac bone osteotomy is considered essential to achieve the above goals in the majority of patients [6,7].

Historically, posterior iliac osteotomy was the earliest type to be introduced. This type of osteotomy was challenging at many levels. It was performed with the patient initially in the prone position and then flipped over to the supine position. Anterior iliac osteotomy includes various modifications but the most common by far is the Salter innominate osteotomy. This involves dividing the iliac bone midway between the anterior superior iliac spine (ASIS) and the anterior inferior iliac spine at the front and the greater sciatic notch (GSN) at the back. The main risk of anterior iliac osteotomy is compressing the neurovascular structures against the inguinal ligament. The oblique iliac osteotomy (also called the diagonal iliac osteotomy) was introduced to overcome the above problem as it divided the iliac bone proximal to the inguinal ligament attachment [1,8-10].

We described here the Y-pelvic osteotomy, which was developed by the Manchester Orthopaedic Group in the United Kingdom [11]. It combines two osteotomies in the shape of the English letter (Y), the oblique iliac L-shaped osteotomy and the posterior iliac bone osteotomy. The former is a complete osteotomy whereas the latter is a partial osteotomy. Y-pelvic osteotomy permits more tissue excursion reducing the tension of the closed abdominal layers. It has less risk to the neurovascular structures, it is associated with less blood loss, and the technique is relatively simple. The osteotomy was named the Y-pelvic osteotomy due to the morphological shape it resembles.

## Technical report

**Figure 1:**
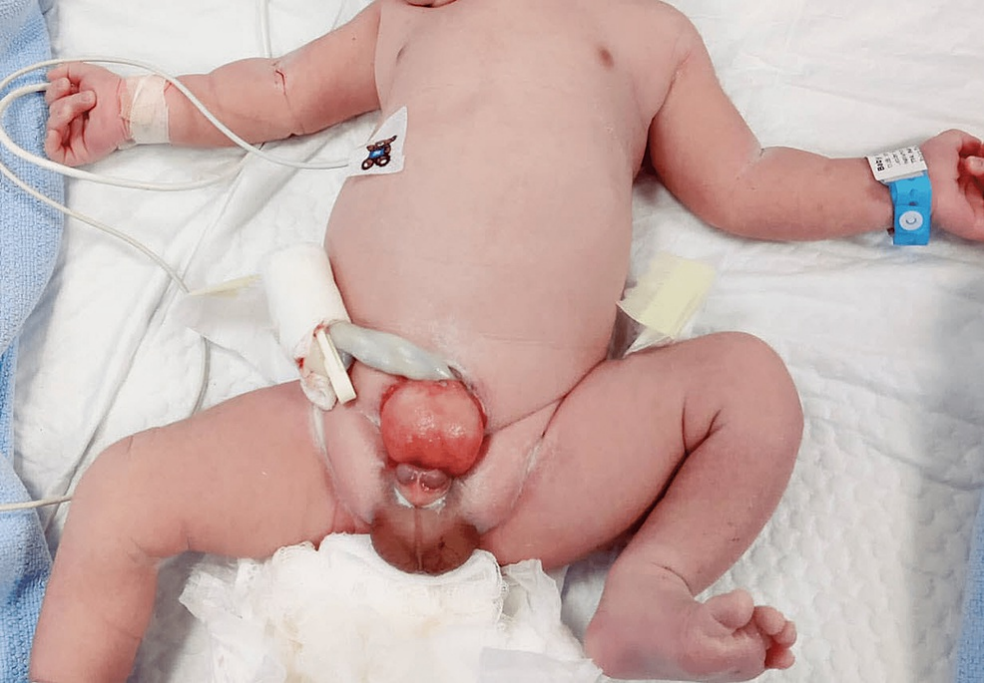
A newborn child with bladder exstrophy. Notice the open abdominal wall and urinary bladder and the underdeveloped genitalia.

The operation is performed under general anesthesia with the child in the supine position. An intravenous line, arterial line, and ECG monitoring are secured by the anesthesia team. A Bovie disposable, single-use grounding pad is applied on the right side of the chest above the costal margin (Figure 1). A warming device should be applied to prevent hypothermia. The surgical field is prepared using chlorohexidine from the costal margin to the feet. Wide-spectrum prophylactic antibiotics are administered at induction.

**Figure 2:**
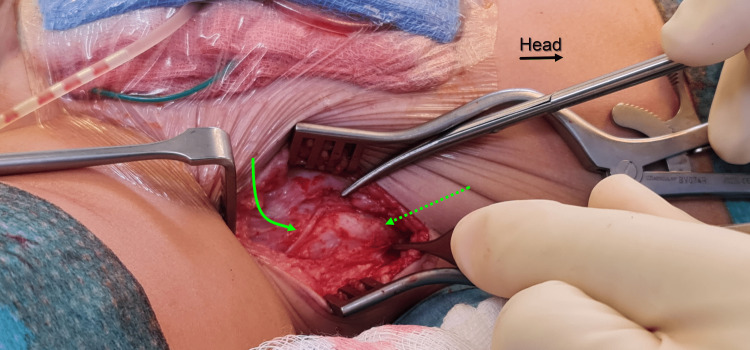
Intraoperative clinical photograph. The abdominal muscles peeled off the iliac apophysis (dashed green arrow). The lateral cutaneous nerve of the thigh is identified and protected through the whole procedure (curved green arrow). Black arrow points to the orientation of the patient and points cranially.

The Y-iliac bone osteotomy is performed after mobilization and reconstruction of the bladder and bladder neck by the urologist. The abdominal wound is covered with wet gauze and Tegaderm. The skin is cleaned again with chlorohexidine to ensure sterility. A sterile rolled towel is placed beneath the ipsilateral buttock after draping. A Smith-Peterson approach is utilized through a bikini groin incision. The incision is made below and parallel to the iliac crest. The latter is more lateral in a patient with BE in comparison to children with dislocated hips. The soft tissue and the abdominal muscles are peeled off the iliac apophysis to expose its outer half only (Figure 2). Then the medial (lower) part of the approach is developed further by identifying and protecting the lateral cutaneous nerve of the thigh.

**Figure 3:**
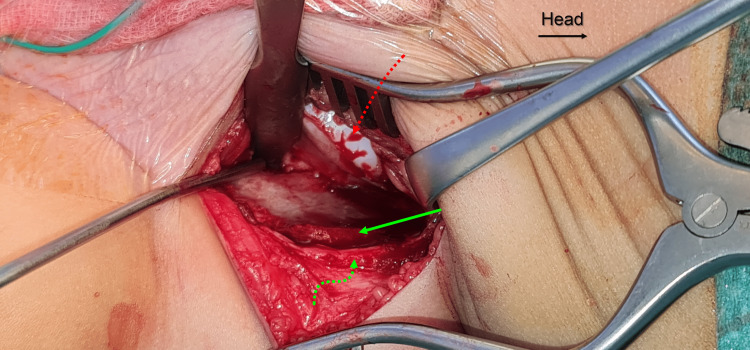
Intraoperative clinical photograph. The photograph shows the exposed inner aspect of the iliac wing. The iliac apophysis is split over the iliac crest (solid green arrow) into a medial half (straight dashed red arrow) and lateral half (green curved dashed arrow).

**Figure 4:**
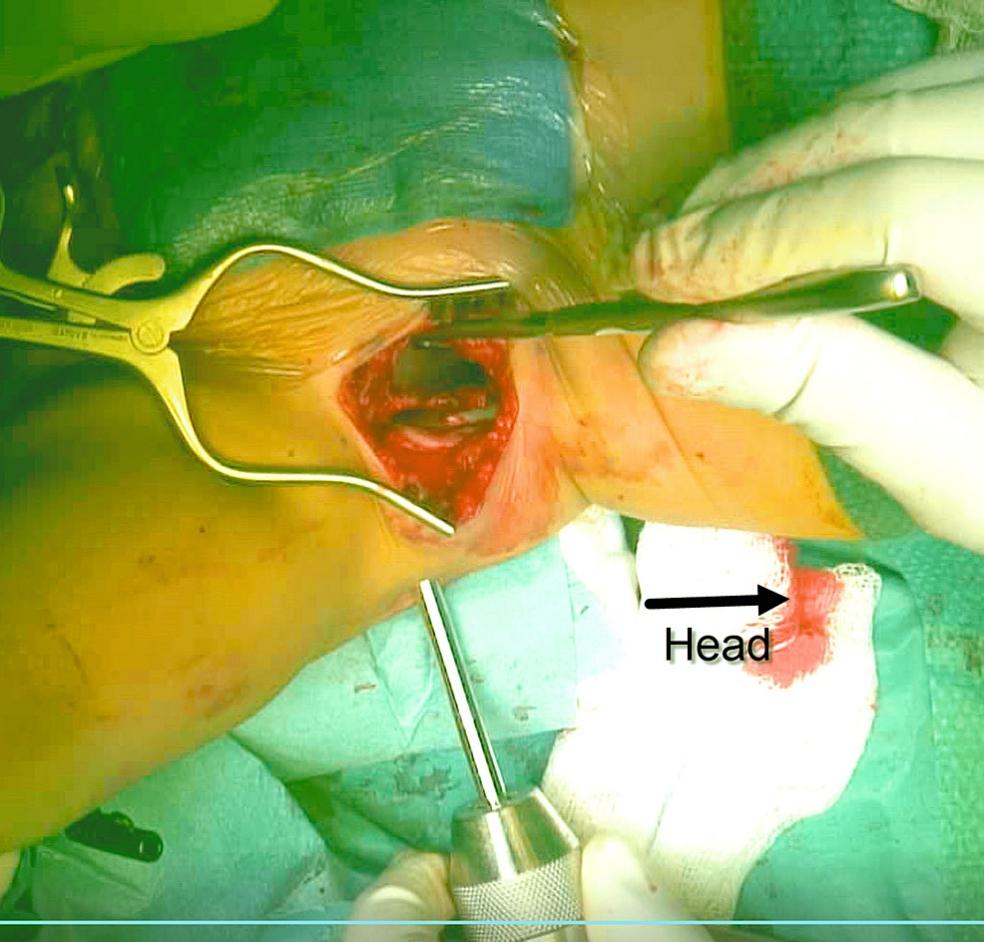
Intraoperative image. The insertion of external fixation half pin under direct vision. The entry point and the exit point should be visible during the insertion. Free hip movement is used as an indicator that there is no hip joint penetration.

The plane between tensor fascia lata and sartorius is developed down to the bony ridge between the anterior superior and inferior iliac spines. The iliac apophysis is split and both sides of the iliac wing are exposed down to the GSN using a periosteal elevator (Figure 3). We do not recommend dividing the psoas or rectus muscle tendons. The external fixator half pin is inserted percutaneously under direct vision from the buttock (Figure 4). We use small or medium external fixator systems depending on the size of the child (Orthofix Medical Inc, Texas, USA). The half pins can be self-drilling and tapping to ease the fixation, but standard half pins can be used as well. We screen the hip clinically to ensure there is no joint penetration. We do not normally use fluoroscopy screening.

**Figure 5:**
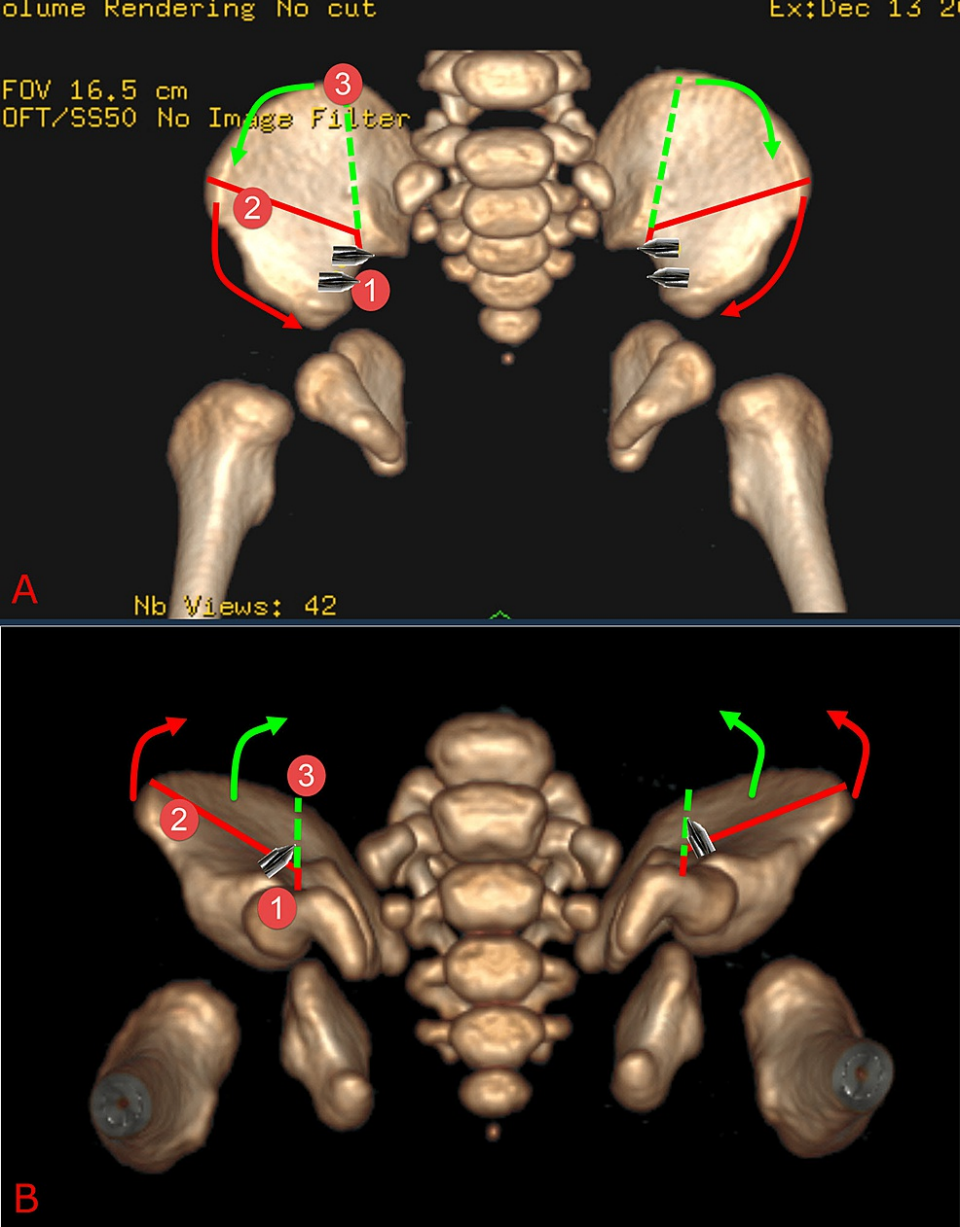
A diagram showing the planned bony cuts of the Y-pelvic osteotomy. Top image (A) is an anteroposterior (AP) view of pelvis CT scan and bottom image (B) is inferosuperior view of pelvis CT scan. The L-shaped bony cut is marked in red and it extends just behind the anterior superior iliac spine toward the pelvis brim then turns forward away from the sacro-iliac joint. The small arm cut is performed first (denoted by red circle 1)  because this cut has to be very precise. Its location prevents inadvertent damage to the sacro-iliac joint or breaching the half pin tracts. The vertical posterior partial (dashed green line) cut extends from the angle of the “L-shaped cut” upward and parallel to the sacro-iliac joint.

The Y-osteotomy consists of two osteotomies: an L-shaped oblique complete osteotomy and a vertical partial osteotomy that runs parallel to the sacro-iliac joint (SIJ) (Figure 5). The first cut is made by the osteotome, and it starts at the pelvic brim, midway between the inserted half pin and the lower part of the SIJ. It extends superior-medial to inferior lateral away from the SIJ to avoid inadvertent damage to the joint. The second cut is made using an oscillating saw and it extends from the iliac crest, 1 cm proximal to the ASIS, toward the top of the first cut. Both (the first and second cuts) should pass at least 5 mm above the external fixator half pins (Figure 5). This is to ensure adequate bone bridge around the pins to reduce the risk of cutting through. The two cuts create the L-shaped osteotomy, which is a complete (full bone thickness) cut. The two fragments should be tested for free movement before proceeding with the third cut.

**Figure 6:**
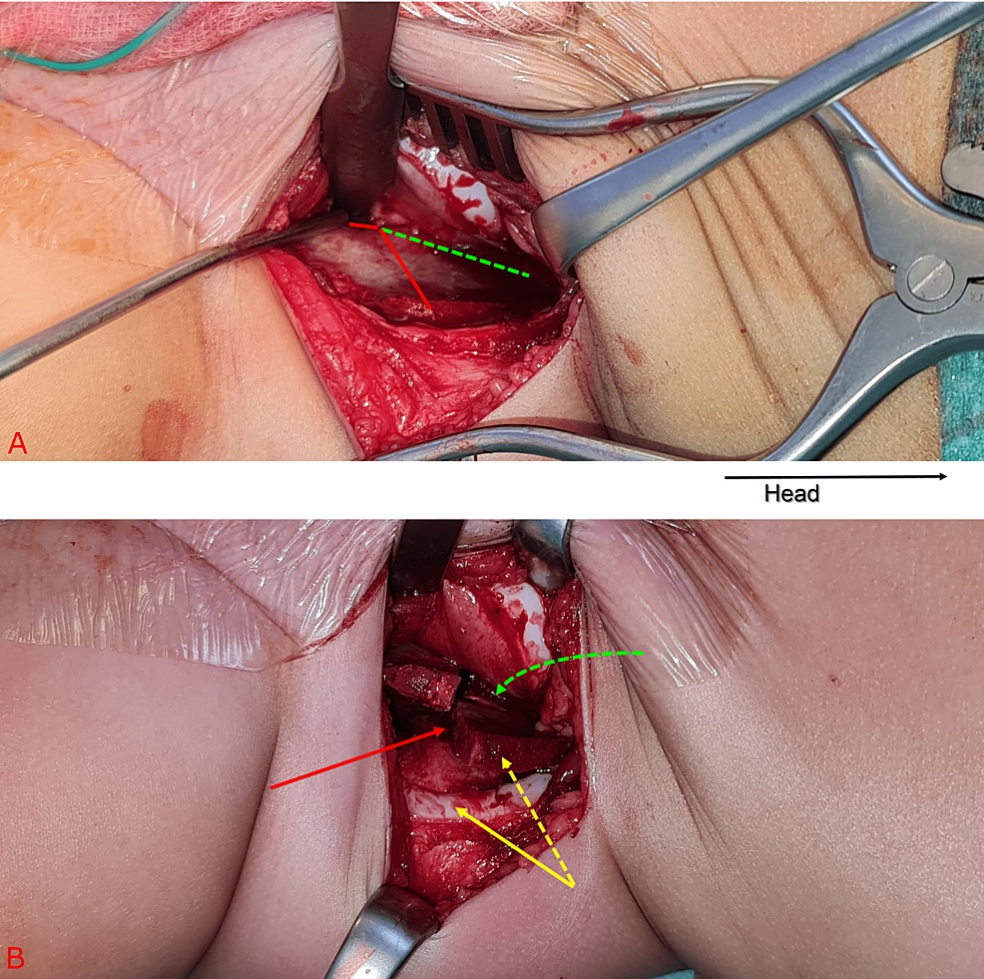
Intraoperative clinical photograph. Top image (A) shows the iliac bone before performing the osteotomies whereas the bottom image (B) shows the iliac bone after performing the osteotomies. The vertical incomplete cut (dashed green line) which is created using an osteotome to score the inner cortex of the iliac bone starting from the angle of the L-shaped osteotomy upward. Two incomplete, parallel and 2 mm apart cuts (like a train track) are made using an osteotome. The 2 mm cortex in-between is then peeled off leaving the outer cortex and periosteum intact. This allows the iliac bone to fold inward (dashed yellow arrow) to allow soft tissue (solid yellow line) to come closer to the midline.

**Figure 7:**
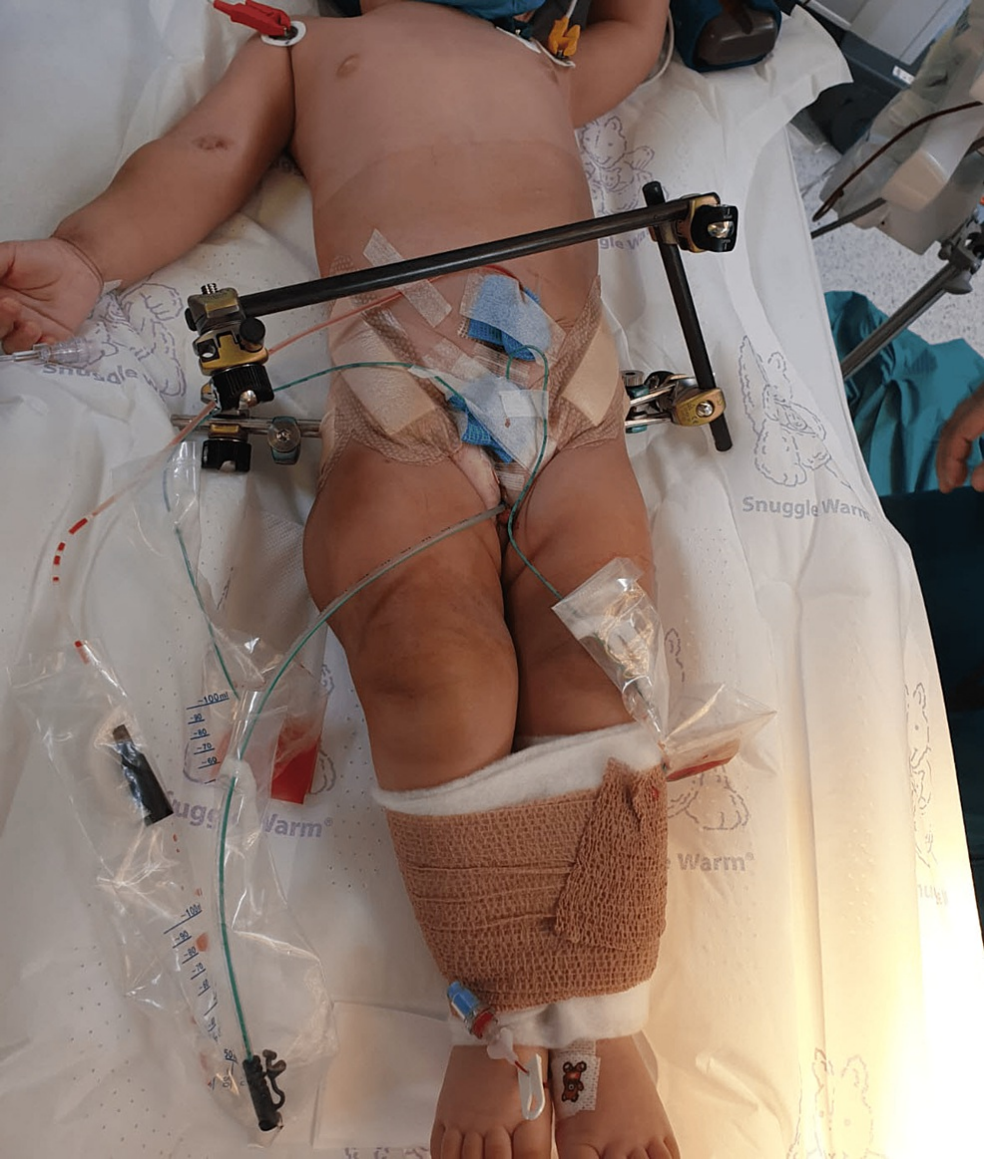
Postoperative clinical photograph. The photograph shows the assembled external fixator and mermaid dressing.

**Figure 8:**
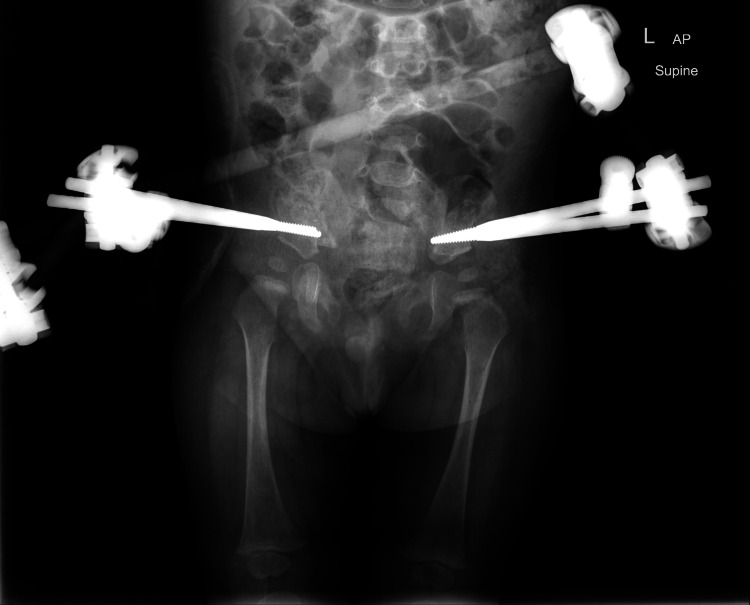
Postoperative plain radiograph. The image shows healed osteotomies with the pins not penetrating the hip joints.

The third cut (or vertical partial osteotomy) extends from the angle of the L-shaped osteotomy upward and parallel to the SIJ. It is placed about 1 cm lateral to the SIJ. It is an incomplete osteotomy where the inner cortex and the cancellous bone only are cut leaving the outside cortex and periosteum intact (Figure 6). This allows the triangular fragment between the two cuts to fold inward. This further allows the pubic rami to come close to each other by allowing the soft tissue to move more toward the midline. The pubic bones can be closed easily following the Y-pelvic osteotomy. The urologist then completes the soft tissue closure by bringing the two pubic bones together using a strong Ticron suture and closing the abdominal wall in layers. The external fixator is then assembled to prevent any tension on the surgical repair. Lower limbs are kept together using the Mermaid dressing (Figure 7). The patient is admitted to a high-dependency unit after surgery for 24-48 hours. Postoperative x-ray is taken at three weeks, and if there is enough callus (Figure 8), the external fixator is removed in the ward. The patient is given painkillers an hour before removal.

## Discussion

Trendelenburg was the first to introduce an orthopedic procedure (bilateral SIJ osteotomy) to facilitate BE closure in 1892. Unfortunately, his first patient died as a result of anemia and complications, and this put a halt on further pelvic osteotomy for BE for a long time [12-14]. Shultz was accredited with the first successful bilateral posterior osteotomy in BE in 1958. The technique immediately gained popularity and became a routine part of BE closure [9,15]. Several shortfalls with the posterior osteotomy technique became apparent and led surgeons to search for a better technique. Posterior iliac osteotomies were performed with the patient in the prone position, which is challenging for the anesthesia team. The thicker iliac bone and soft tissue covering posteriorly caused increased blood loss and postoperative pain. Myelomeningoceles are not uncommon in cloacal exstrophy. The incision for posterior iliac osteotomy can be very close to the myelomeningocele closure, which could lead to skin problems. After osteotomies are completed, the patient must be flipped over to the supine position for the urogenital reconstruction which is not free of risk. This also means that osteotomies fine-tuning to allow tension-free closure is not possible or at least more complicated.

Several authors proposed performing pubic bone osteotomy through the same incision of the urological repair. The bones are accessible, there is less blood loss, and it is technically easy. It was also proposed that the osteotomy could be performed by a urologist without the need for an orthopedic surgeon. Although this allows the two ends of the pubic bones to come very close together, the technique was not successful in providing an effective way to reduce the tension from surgical closure. This is partly because the abdominal muscles are attached laterally to the pubic bones [16]. Interestingly, there has been a recent interest in reviving this technique [17-19].

The innominate osteotomy was popularized by Salter to treat congenital dislocation of the hip [20]. It was then utilized in failed or revision BE repair. It was then extended successfully for primary closure [21-25]. The main problem with the innominate osteotomy is that the bony cut and subsequent rotation comprises the space available for the femoral sheath and its contents including the femoral nerve and vessels. 

Sponseller and colleagues introduced the “combined approach” for older children by adding an incomplete vertical posterior iliac osteotomy [26]. The purpose of the vertical incision is to compensate for the loss of elasticity of the SIJ in older children. Unfortunately, the use of this combined procedure has not eliminated the risk of nerve injury. Segev reported three nerve injuries in four patients. Although two had a complete recovery, the third continued to suffer from foot drop [27].

The Y-pelvic osteotomy overcomes the problems that current techniques have. It is posterior to the inguinal ligament attachment; therefore, there is a very low risk of compressing the neurovascular structures within the inguinal sheath that could occur in an innominate Salter pelvic osteotomy. Moreover, the abdominal wall muscles are attached more posteriorly to the iliac wings. Anterior osteotomy alone is not effective to bring these muscles closer together anteriorly for closure without tension. The Y-pelvic osteotomy allows the attachments of the abdominal muscles to move forward and medially to facilitate closure. The first cut is through the thinnest part of the iliac bone and the vertical cut is not complete. Both do not cause excessive blood loss in comparison to other techniques.

## Conclusions

The proposed "Y-type" pelvic osteotomy combines the benefit of the oblique (diagonal) pelvic osteotomy in protecting the neurovascular structures in the femoral sheath, and the posterior pelvic osteotomy to provide a more efficient way to mobilize the lower abdominal wall muscles. Although it was initially introduced for patients with symphysis diastasis of more than 6 cm, it has become a standard procedure in treating our patients with BE because it is simple, effective, bleeds less, and takes just a little extra time (around 5 minutes more) than the oblique osteotomy alone. Our urologist colleagues believe that the Y-pelvic osteotomy allows an easier closure with less tension on the abdominal structure in comparison to the previously used osteotomies.

The above-stated advantages are currently being evaluated in relation to important clinical outcomes including hemoglobin drop, blood transfusion rate and volume, length of stay, and dehiscence rate. These will be the subject of our next publication. Given the complexity of the condition and the multiple factors that can influence the above clinical outcomes, a large number of patients are required to demonstrate the value of the Y-pelvic osteotomy. 
